# Ultra-Short Laser-Assisted Micro-Structure Formations on Mg/Zn Double-Doped Calcium Phosphate Ceramics for Enhanced Antimicrobial Activity

**DOI:** 10.3390/ma16206626

**Published:** 2023-10-10

**Authors:** Albena Daskalova, Kostadinka Sezanova, Liliya Angelova, Tsvetelina Paunova-Krasteva, Rumiana Gergulova, Daniela Kovacheva, Diana Rabadjieva

**Affiliations:** 1 Institute of Electronics, Bulgarian Academy of Sciences, 1784 Sofia, Bulgaria; albdaskalova@gmail.com (A.D.); lily1986@abv.bg (L.A.); 2 Institute of General and Inorganic Chemistry, Bulgarian Academy of Sciences, 1113 Sofia, Bulgaria; ksezanova@abv.bg (K.S.); rumigg@yahoo.com (R.G.); didka@svr.igic.bas.bg (D.K.); 3 Institute of Microbiology, Bulgarian Academy of Sciences, 1113 Sofia, Bulgaria; pauny@abv.bg

**Keywords:** doped tricalcium phosphate, ceramic tablets, femtosecond laser processing, surface modification, antimicrobial characteristics

## Abstract

Bacterial infection is one of the most common and harmful medical issues following the implantation of materials and devices in the body leading to antibiotic resistance of diverse bacterial strains. In this work, a novel approach is presented combining adopted laser-based patterning method in addition to doping (Mg and Zn) metal ions to prepare calcium phosphate ceramic substrate, applicable in medicine, with enhanced surface antimicrobial characteristics. The preparation of tablets containing Mg (8.44 mol%) and Zn (2.63 mol%) β-tricalcium phosphate involved biomimetic precipitation of amorphous calcium phosphate in media of simulated body fluid enriched with Mg^2+^ and Zn^2+^ ions as well as the presence of valine as an organic additive, followed by step-wise calcination up to 1000 °C. The results from laser processing showed formation of deeper patterns with increased surface roughness (from 4.9 µm to 9.4 µm) as laser power and velocity increase, keeping constant the hatch sizes of 50 µm. The textured surfaces consist of peaks and valleys arrangement that change the morphology of *Escherichia coli* cells and decrease of cell viability. Our study reveals the possibilities of the application of ultra-short laser radiation as a potential alternative therapy for controlling the antimicrobial effect of the ceramic surface.

## 1. Introduction

The discovery of alternative materials with enhanced antimicrobial characteristics that will help decrease the use of antibiotics has become a priority in developing innovative solutions for fighting the increasing antibiotic resistance of diverse bacterial strains. In the field of orthopedics, implant replacement surgery is becoming more common, especially as society becomes older. One of the most frequent and dangerous medical problems following device implantation is bacterial infection. The ubiquitous use of antibiotics to treat it leads to the development of antibiotic-resistant strains. Thus, the search for new methods or combinations of several methods for more effective antibacterial protection of implants and devices is the subject of increasing research interest.

Calcium orthophosphates are non-toxic biocompatible substances possessing osteoconductive properties, which allow them to be implanted in the body, since they do not cause an adverse immune response [1]. Moreover, their major ionic composition and crystal structure are similar to these of biological apatite. One of the main characteristics of biological apatite is that it contains small amounts of incorporated Na^+^, K^+^, Mg^2+^, Zn^2+^, Sr^2+^, etc., ions [1,2,3]. Therefore, interest in the synthesis of ion-substituted calcium phosphate materials approaching the composition of hard tissues has increased. The aspiration of scientists is to obtain materials with improved bioadaptable properties. Zn-doped hydroxyapatite (HA) has been shown to have a better antibacterial effect and osteoblastic proliferation activity [4]. Sulfonated 2 poly(etheretherketone)/(Sr and Ce) co-substituted composite coating of HA possesses increased corrosion resistance and antibacterial activity [5]. In mesenchyme stem cells derived from human bone marrow, Mg-doped biphasic calcium phosphate nanoparticles containing silver showed great cytocompatibility [6]. Zn- [7] or Sr-, Zn- and Mn- [8] containing β-tricalcium phosphate (β-TCP) have been shown to favor the self-repair of the bone. Mg and Zn are preferable as substituents since they are essential for the organism, playing an important role in the formation and growth of the skeleton [9,10]. Mg is essential to living cells such as osteoblasts and osteoclasts [11]. Moreover, deficiency of both Mg and Zn contributes to the development of osteoporosis [11,12].

The main types of calcium-orthophosphate-based biomaterials, differing in preparation techniques and physical–mechanical properties are calcium-phosphate ceramics, cellular and tissue scaffolds, cements, composites and coatings on metal implants improving their surface properties and promoting cellular orientation, adhesion and proliferation. Calcium orthophosphate ceramics, which are already actively used in medicine, are usually based on HA, α- and β-tricalcium-phosphates (TCP) or multiphase formulations based on them [2,3]. One of the most important qualities of ceramic calcium-phosphate-based bone implants is to be resorbable, which requires their rate of dissolution to match the rate of new bone formation [13] to ensure that they will be efficiently absorbed by the body. Solubility diagram of the system Ca(OH)_2_-H_3_PO_4_-H_2_O at 37 °C, prepared by thermodynamic calculations [14], show that at physiological pH of 7.2–7.4, HA possesses the lowest solubility among all other calcium phosphates, followed by β-TCP (with an order of magnitude difference). The solubility of α-TCP is several orders of magnitude higher. This defines HA ceramics as bioactive, i.e., they stimulate the formation of new tissue on the surface but are not resorbed. From this point of view, β-TCP bioceramics are attractive materials for bone reconstruction and remodeling. Moreover, the degree of dissolution of β-TCP is close to that of bone apatite [15].

The surface topography of calcium phosphate ceramic materials, scaffolds or coatings is of primary importance for osseointegration processes, since they participate in bone-bonding ability and enhance new bone [2,16]. Different techniques were developed for surface treatment to improve its osteogenic and antibacterial properties [16]. The classical method is incorporation of ions with antibacterial effects, such as Ag^+^, Cu^2+^ or Zn^2+^ [16,17]. The main disadvantage of this method is the concentration limit, especially for Cu and Zn, above which they can cause harm to the organism. Another type of chemical processing of the surface is with acid–alkali solutions, polymer-containing systems, etc. [17,18]. Surface roughness is a key factor in cell proliferation and growth and as well as in increases in antibacterial activity [16,19,20]. Sandblasting, polishing with SiC papers, acid etching, plasma spring or anodizing is usually used [20,21]. The problem is that more than one processing technique is required, and contamination is possible. As an alternative to conventional methods, the structuration of surfaces by ultrashort-pulsed (femtosecond) lasers introduces contactless patterning of the surfaces, created in a single step, with improved long-term stability of the created structures [22,23]. Successful attempts were performed by Faria et al. [24], by utilizing a hybrid laser-assisted approach for material functionalization of HA-coated zirconia, whereas a surface-patterning technique will prevent the detachment of ceramic coatings. The adopted laser-based approach for producing the functionalized surfaces demonstrates its efficacy in the creation of reproducible micro-textured surfaces coated with the bioactive phase, which assured bioactive retention and non-degradation [24].

Ultra-short laser–matter interactions have become a useful tool for gentle material processing due to the nature of the interaction process in the fs time scale [25,26]. The energy deposition in this regime is highly localized with the capability to ionize a large number of electrons [27]. The surface characteristics of the ceramic biomaterial influence the cell-based interactions, protein adherence, and antimicrobial properties, thus affecting the osseointegration process [28,29]. Therefore, the implant surface topography and chemistry have to be considered when designing the ideal scaffolds. In this study, we will present the combination of laser-induced surface texturing with an examination of doped β-tricalcium phosphate ceramic for the creation of orthopedic implants, to achieve improved biological efficiency and antibacterial activity. Exploring the impact of the laser processing parameters on the efficiency of the obtained patterns has been the focus of numerous studies aimed at minimizing the heat-affected zones and optimizing surface roughness for diverse applications in biomedicine. Research efforts have been made to define the best conditions to process brittle ceramic material with different experimental approaches. Reports on the application of femtosecond laser radiation to process bioceramic material are scarce. However, there are several research efforts that prove the efficacy of the method. For example, Zhang et al. [30] reported successful machining of zirconia (ZrO_2_) ceramics utilizing the pump-probe imaging method. They were able to obtain precision processing in the laser drilling of zirconia ceramics. Chen et al. [31] studied the influence of femtosecond laser parameters on laser ablation of Al_2_O_3_ ceramics. They found that the phase transition develops from solid to vapor without the initiation of melting, thus leading to precise, well-defined micrometer-sized structures.

In this research, a systematic study of diverse patterning designs, showing the dependence of surface characteristics in relation to antimicrobial effects, allows definition of reproducible laser patterning models and eventual achievement of best conditions for microbial biofilm rupture. The aim of this paper is a comparative experimental study of the surface modification of biomimetically synthesized Mg and Zn double-doped β-tricalcium phosphate ceramic, textured by a femtosecond laser to evaluate different processing modes. In addition, a preliminary evaluation of *Escherichia coli* viability and changes in cell morphology were performed on the selected samples to demonstrate that the implementation of laser irradiation for producing surfaces with increased percentage of roughness in addition to doping metal ions could potentially lead to formation of nano- and micro-scale or hybrid surface structures with a potential antimicrobial effect.

## 2. Materials and Methods 

### 2.1. Synthesis of Mg/Zn Double-Doped β-TCP and Preparation of Ceramic Tablets

Ceramic tablets of Mg/Zn double-doped β-TCP were prepared by a three-step method: (i) biomimetic synthesis of amorphous (Mg, Zn)-modified calcium phosphate; (ii) tablet preparation; and (iii) calcination of the tablets.

A method of continuous precipitation was used to synthesize amorphous (Mg, Zn)-modified calcium phosphate. Calcium- and phosphorus-free conventional simulated body fluids [32], enriched with valine (Sigma-Aldrich, St. Lois, MO, USA, A.R.) were used as solvents for K_2_HPO_4_, (Merck, Darmstatd, Germany A.R.), CaCl_2_·2H_2_O (Sigma-Aldrich, A.R.), MgCl_2_·6H_2_O (Merck, A.R.) and ZnCl_2_·2H_2_O (Merck, A.R.) (Table 1). 

Combined apparatus for automatic titration and controlled synthesis (Titrando 907, Metrohm AG, Herisau, Switzerland) was used. All reagents (Solution 1, Solution 2 and Solution 3, Table 1) were added to a buffer solution (Table 1) at room temperature and continuous stirring at a rate of 3 mL·min^−1^ keeping the pH at 8.0–8.2 using 0.05 M KOH. The pH of Solution 3 (Table 1) was not corrected to avoid Zn-hydrolysis products. 

Concentration of dopants were selected to achieve Mg/(Ca + Mg + Zn) of 7 mole % and Zn/(Ca + Mg + Zn) of 3 mole %. In the syntheses, the Mg concentration was doubled.

After 2 h of precipitation, the suspension was matured in the mother liquor for 1 h under continuous stirring at room temperature and then washed with water (to remove chlorides) and lyophilized.

The lyophilized powder was milled in a ball mill (Fritsch 6) for 0.5 h at 500 rpm, then passed through a 0.1 mm sieve. An amount of 0.65 g of the sieved powder was tableted with a pressure of 6.5 tons for 2 min on a SPECAC GS15011 press (Orpington, GB). Prepared tablets with a diameter of 13 mm were calcined at 200, 400, 600, 800 and 1000 °C and atmospheric pressure in high-temperature furnace (type VP 04/17, LAC Ltd. Company, Rajhrad, Czech Republic). The working regime was heating at a rate of 3 °C·min^−1^ until the desired temperature was reached and then keeping it constant for 3 h.

### 2.2. Processing with Femtosecond Laser 

The patterned rows were acquired with linearly polarized laser pulses on Mg/Zn double-doped β-TCP disks using a Solstice Ace system, delivering laser pulses with a pulse duration of 70 fs, wavelength of λ = 800 nm and repetition frequency of ƒ = 1 KHz at an average output power of the laser system up to 6 W. The focus spot diameter is 25 μm at 1/e^2^ of its intensity maximum at Gaussian-profile intensity distribution. The samples were positioned in air on an XY translation stage orthogonal to the laser beam. The motion stage was then rasterized at diverse scanning speed, generating lines over the sample surface. The energy delivery was controlled via a polarizing beam splitter. The sample stage scanning velocity (V) was varied between 0.2 mm/s and 15 mm/s. Different laser powers (P) and distances between separate rows (d_x_), designed in the form of a mesh created by laser raster scanning of the surface in two perpendicular directions, were applied.

### 2.3. Characterization 

#### 2.3.1. Chemical Analysis 

The sum of Ca^2+^, Zn^2+^ and Mg^2+^ ions in the solid samples was determined complexometrically with EDTA at pH 10. The concentrations of Zn^2+^ and Mg^2+^ ions were determined by ICP-OES (PRODIGY 7, Teledyne, Leeman Labs, Hudson, NH, USA). P-PO_4_^3−^ and Cl^−^ ions were analyzed spectrophotometrically by NOVA 60 equipment (Darmstatd, Germany) using Merck and Spectroquant test kits. 

#### 2.3.2. XRD Analysis 

Powder X-ray diffraction (PXRD) was performed using a Bruker D8 Advance diffractometer with Cu Kα radiation and a LynxEye detector (Bruker AXS Advanced X-ray Solutions GmbH, Billerica, MA, USA). For the primary phase identification, the data were collected in the range of 10 to 90° 2θ with step 0.03° 2θ, with counting time 57 s/step. The phase composition was identified using the ICDD-PDF2 (2014) database. 

The Rietveld structure refinement was performed by using Bruker Topas v.4.2 program in order to proof substitution of Ca from Mg and Zn in the crystal structure of β-TCP. Powder diffraction patterns were collected at room temperature within the range of 5 to 120° 2θ with a step of 0.02° 2θ and 175 s/step counting time and a sample rotation of 15 rpm.

#### 2.3.3. Confocal Microscopy

For surface and roughness evaluation of the laser-structured or control ceramic pellets, 3D confocal images were obtained by a 3D μsurf explorer confocal microscope (Nano-focus, Oberhausen, Germany). The apparatus provides high-resolution images of the surface. For 3D visualization of the confocal images obtained, ProfilmOnline software Version 3.0.1 (https://www.profilmonline.com) was used (accessed on 30 March 2023). Additional roughness analysis was performed; values of the Sq (the extension of the mean value of the deviations of the surface height from the median line to a surface area) roughness parameter were obtained as average values over 5 separate measurements in accordance with the ISO 4287 standard [33].

#### 2.3.4. Raman Spectroscopy 

The effect of the laser processing on the phase of the ceramic material was evaluated by Raman spectroscopy (Raman spectrometer Horiba MicroRaman, Kyoto, Japan). The Raman spectra were obtained using a 532 nm laser as a light source, and all reported spectra were averaged over five measurements, acquired in different points on the same sample.

#### 2.3.5. Scanning Electron Microscopy (SEM) Analysis of Tablet Morphology and Microstructure

The morphology and microstructure of the annealed tablets was investigated using a Lyra I XMU Scanning electron microscope (Tescan, Czech Republic), while the femtosecond laser-treated tablets were investigated using JEOL JSM 6390 (Tokyo, Japan) apparatus. The samples were gold-sputtered (~20 nm Au layer) in vacuum, and SEM images were taken at several different magnifications. 

#### 2.3.6. Estimation of Bacterial Viability by Plate Count

The bacterial strain used in the study, *E. coli* 25922, was obtained from the American Type Culture Collection (ATCC). It was stored in 8% DMSO at a temperature of −80 °C. Prior to the experiments, the strain was inoculated in tryptic soy broth (TSB, Sigma-Aldrich, Berlin, Germany) and maintained at 4 °C on tryptic soy agar (TSA, Sigma-Aldrich, Germany) slants.

The ceramic tablets were pre-treated by dry sterilization at a temperature of 200 °C for 30 min. The antimicrobial activity was determined according to a standard evaluation test of antimicrobial surface designs with minor modifications [34]. A volume of 2 mL of overnight 1:100 diluted bacterial inoculum was co-cultivated with sterile ceramic tablets. After a cultivation period of 24 h at 37 °C, bacteria were collected, diluted in phosphate-buffered saline (PBS) and calibrated to 0.5 McFarland units (approximately 1 × 10^8^ colony forming units CFU/mL) by using a Densilameter II (Microlatest). The number of surviving bacteria (CFU) was counted by transferring 100 μL of bacterial suspension onto TSA in a petri dish, followed by incubation for 24 h at 37 °C. The assay was performed in duplicate. 

#### 2.3.7. Evaluation of *E. coli* Cell Morphology by Scanning Electron Microscopy (SEM)

For the purpose of evaluating the SEM investigation, the bacteria were incubated overnight (18 h) in TSB at 37 °C. An overnight bacterial inoculum was prepared after 1:100 dilution in TSB. The diluted inoculum was then resuspended and placed in a 12-well plate, with 2 mL of the inoculum added to each well in the presence of sterile ceramic tablets. Duplicate samples were prepared for each variant. The plate was incubated at 37 °C in a static condition for 24 h. After this interval, the laser-treated tablets were washed and fixed with 4% glutaraldehyde in 0.1M Na cacodylate buffer at 4 °C for 2 h. The tablets were then washed again in cacodylate buffer and post-fixed for 1 h using a solution of 1% OsO_4_ at 4 °C. After washing twice with cacodylate buffer, a dehydration procedure was performed in a graded ethanol series (30, 50, 70, 80, 90 and 100%) through 15 min time intervals. Finally, the dehydrated tablets were installed on scanning electron microscopy holders and sputter-coated with a layer of gold using a vacuum evaporator (Edwards, Stansted, UK). The observations of the bacterial morphology were made on Lyra I XMU Scanning electron microscope (Tescan, Czech Republic) with an accelerating voltage of 20 kV.

## 3. Results 

### 3.1. Synthesis of Mg/Zn Double-Doped β-TCP and Preparation of Ceramic Tablets 

**Figure 1:**
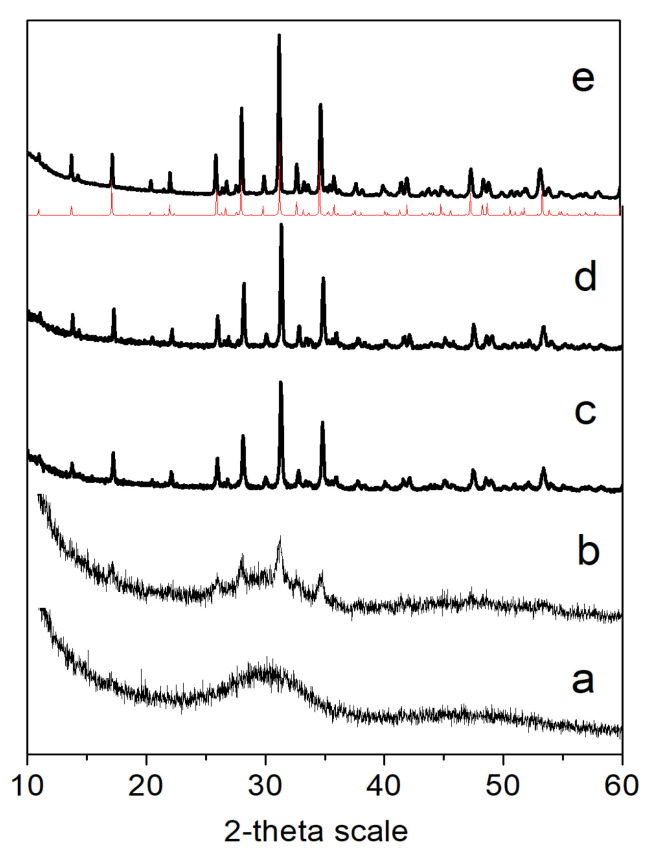
XRD studies of the initial (**a**) and the calcined at different temperature tablets: (**b**) 400 °C; (**c**) 600 °C; (**d**) 800 °C and (**e**) 1000 °C. Red line—Ca_3_(PO_4_)_2_—PDF # 09-0169.

The chemical and XRD analysis of biomimetically precipitated powder showed the formation of amorphous calcium phosphate (Figure 1a) with (Ca + Mg + Zn)/P = 1.56 and concentration of Mg 8.44 mole % and Zn 2.63 mole %. In addition, 4.3 mmolCl·g^−1^, and less than 0.036 mmolNa·g^−1^ and 0.06 mmolK·g^−1^, were detected. After calcination, the amorphous products were transformed into Mg/Zn doped β-TCP (Figure 1e). The transformation started at 400 °C (Figure 1b) and was fully completed at 600 °C (Figure 1c). 

In order to determine the crystal structure of the substituted phase, a Rietveld refinement method was used. The best results were obtained under the assumption that Ca is replaced by Zn and Mg in the Ca(5) position in the structure of β-TCP [35]. The calculated unit cell parameters of samples calcined at 1000 °C reveal a decrease in their values in comparison with the unsubstituted β-TCP (Table 2), which is evidence that Mg and Zn are included in the crystal structure of β-TCP.

**Figure 2:**
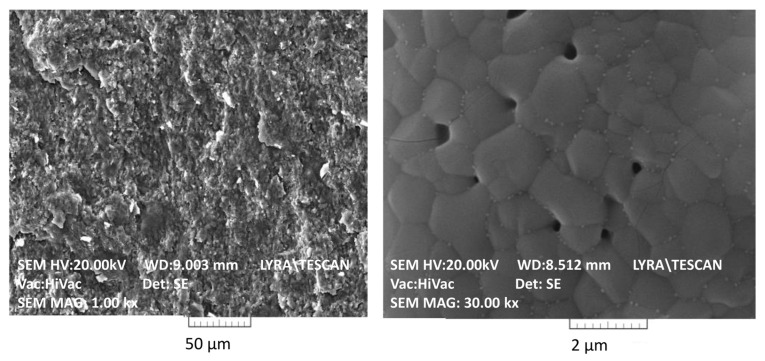
SEM images (at ×1000 [left] and ×30,000 [right] magnification) of ceramic pellets from Mg/Zn-β-TCP calcined at 1000 °C.

The calculated geometric density of the amorphous calcium phosphate tablets was 2.21 g·cm^−3^, which decreased by 32% (to 1.50 g·cm^−3^) upon calcination. This significant volume contraction leads to the formation of a dense ceramic structure as seen in the SEM images (Figure 2).

### 3.2. Processing with Femtosecond Laser 

Ultra-short laser texturing was successfully applied to doped CaP substrates without monitoring changes in the ceramic microstructure. The laser modification induces selective material removal, thus inducing a gentle modification of the irradiated zone of the brittle ceramic material.

In order to evaluate the best texturing conditions, a precise selection of the laser parameters is required to create surface patterns with high quality. 

**Figure 3:**
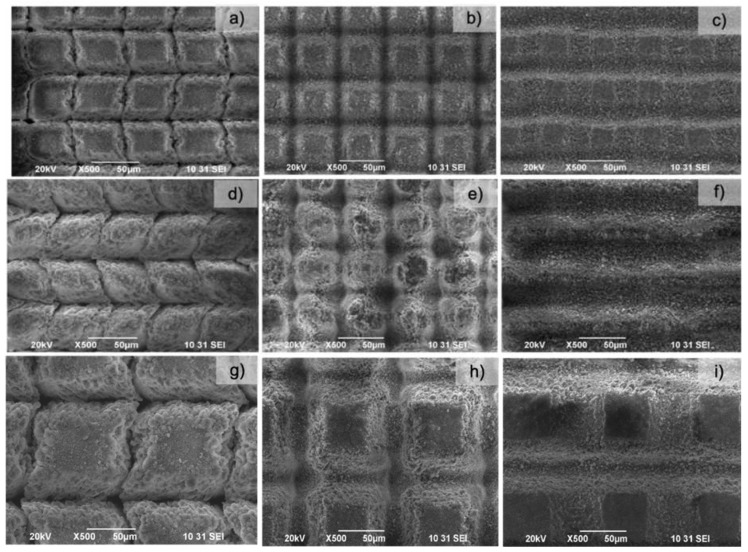
SEM images (at ×500 magnification) of laser-treated pellets with laser power (P) of 20 mW (**a**–**c**), 60 mW (**d**–**f**), and 100 mW (**g**–**i**), with distance between separate rows (d_x_) = 50 μm and velocity 0.2 mm/s (**a**,**d**,**g**); 1.5 mm/s (**b**,**e**,**h**); and 7.5 mm/s (**c**,**f**,**i**).

Figure 3 shows the change in surface morphology with changing laser parameters. Obtained results demonstrate a decrease in surface roughness by increasing scanning velocities (V) at constant laser power (P) and distance between separate rows (d_x_). 

**Figure 4:**
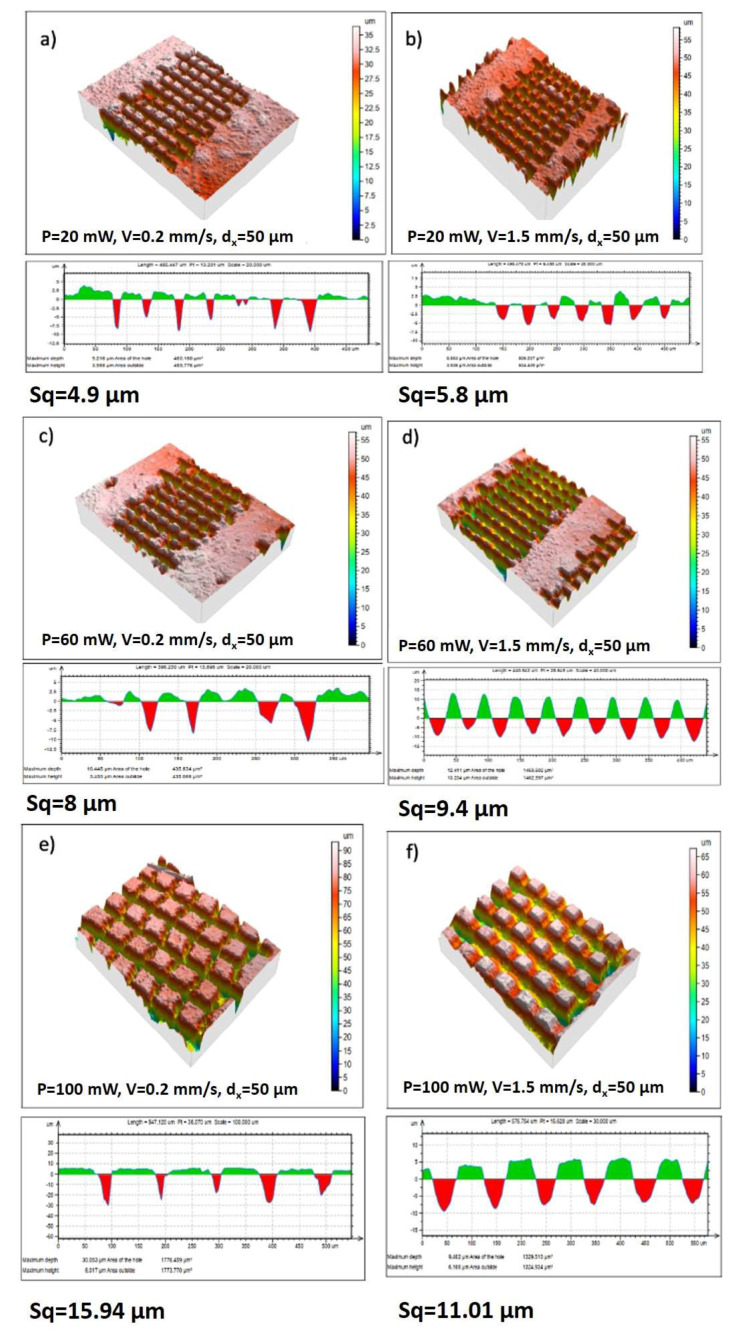
3D confocal microscope (CLSM) images (at ×20 magnification) of femtosecond laser-treated pellets by different power and velocity: (**a**) P = 20 mW, V = 0.2 mm/s; (**b**) P = 20 mW, V = 1.5 mm/s; (**c**) P = 60 mW, V = 0.2 mm/s; (**d**) P = 60 mW, V = 1.5 mm/s; (**e**) P = 100 mW, V = 0.2 mm/s; (**f**) P = 100 mW, V = 1.5 mm/s.

By adjusting the laser scanning parameters, line- and cross-like patterns with diverse row depths were produced via changing the scanning velocities and the applied laser power. We observed a decrease in the pattern depth with scanning velocity increment. The morphological analysis demonstrates the formation of different granular and porous microstructures without evidence of cracks or quality deterioration. The laser-treated and non-treated (Mg/Zn)-β-TCP surfaces presented different surface morphologies (Figure 3a–f). The (Mg/Zn)-β-TCP surface treated with lower laser power (P = 20 mW) (Figure 3a–c) possesses slightly smoother structures. Furthermore, with increasing P to 60 mW (Figure 3d–f), a grain deposition of ablated material resembling on the top of and between the created microstructures was observed for V between 0.2 mm/s and 1.5 mm/s. The 3D confocal analysis (Figure 4) complements the SEM results and estimated relation between scanning speed, laser power and depth of the ablated craters.

It was found that increasing the depth of the single pattern was strongly influenced by the laser power. Crater width increased with increasing laser power, while increasing scanning speed to 1.5 mm/s led to a change in crater forms, from V- to U-shaped (Figure 4a,d). The surface roughness was assessed through an evaluation of the arithmetic average roughness (Sq) from the profiles obtained through confocal microscopy. A cross-sectional profile of the created structures is also presented. However, the increase in scanning velocity did not alter the repeatability of the patterns produced. 

The textures represent highly ordered rectangle-like, V-shaped micropillars (in the case of V = 0.2 mm/s for P = 20 and 60 mW—Figure 3b,e) due to the laser raster scanning pattern used in two perpendicular directions, so a grid design of the surface is created. When a further increase in the scanning velocity is performed (V = 1.5 mm/s), the depth of the created microchannel around the created rectangles decreases, and the formation of clear pillar-like features diminishes (Figure 3d,f). The grid microporous structure is not well defined, nor are the channel depth and edges; no ejected material in the form of micropillars is observed, due to the increase in the scanning velocity applied and, as a consequence, more gentle laser-material interaction is observed. All of the structures obtained are highly and precisely reproducible and controlled by the precise selection of the applied laser parameters.

**Figure 5:**
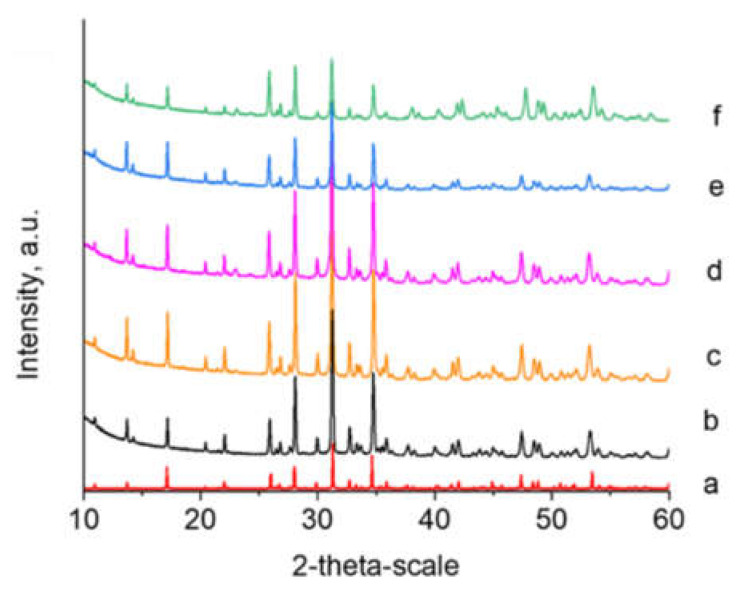
XRD studies of (Mg/Zn)-β-TCP ceramic pellets (**a**) standard Ca_3_(PO_4_)_2_—PDF # 09-0169; (**b**) without laser modification; and laser-treated at (**c**) P = 20 mW, V = 1.5 mm/s, d_x_ = 50 μm; (**d**) P = 60 mW, V = 1.5 mm/s, d_x_ = 50 μm; (**e**) P = 100 mW, V = 1.5 mm/s, d_x_ = 100 μm; (**f**) P = 100 mW, 15 mm/s, d_x_ = 100 μm.

**Figure 6:**
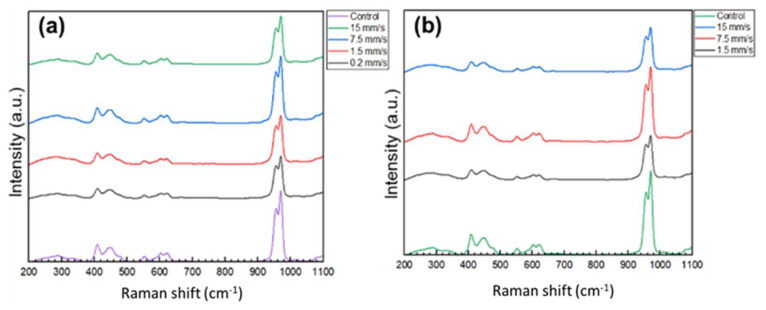
Raman spectra of the (Mg/Zn)-β-TCP ceramic pellets ablated in a grid pattern at (**a**) P = 20 mW, V= 0.2, 1.5, 7.5, 15 mm/s, d_x_ = 50 μm; and (**b**) P = 60 mW, V = 1.5, 7.5, 15 mm/s, d_x_ = 50 μm.

Both XRD (Figure 5) and Raman analysis (Figure 6) did not show the presence of α-TCP or other high-temperature calcium-phosphate phases after laser modifications with variation of scanning speed and applied laser power. As can be seen from Figure 5, the presence of β-TCP in all laser-processed and control pellets was confirmed by the reflection in accordance with standard Ca_3_(PO_4_)_2_—PDF # 09-0169 (ICDD-PDF2 (2014) database).

However, a decrease in V to 0.2 mm/s, P = 20 mW led to a slight decrease in the intensity of the Raman signal (Figure 6), but no change in the form or position of the peaks was observed after laser ablation of the ceramic material with respect to control surfaces. The complementary results obtained by the XRD and Raman analyses indicate once again that the femtosecond laser surface structuring of the (Mg/Zn)-β-TCP pellets is a contactless, finely controlled method that allows precise tuning of surface micromorphology without significantly affecting the crystallinity and phase of the ceramic material.

### 3.3. Analysis of an Antimicrobial Effect

**Figure 7:**
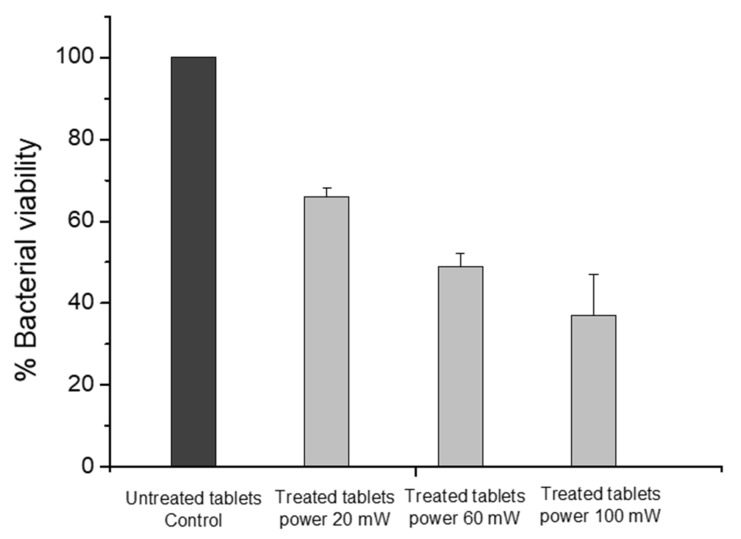
Assessment of the viability of bacterial cells co-cultivated with laser-treated ceramic tablets at three different laser powers (P = 20, 60, 100 mW) and scanning velocity of V = 1.5 mm/s when compared to the control samples (untreated tablets). Colony-forming units (CFUs) were counted and represented as a percentage of viable cells in comparison to colonies obtained from untreated tablets. The control samples were accepted as 100% and the other values were normalized to it.

To evaluate the viability of *E. coli* bacterial cells co-cultured with the sterile ceramic tablets, we applied the classic agar plating method. The plate-counting measurements verify the antimicrobial activity of the treated tablets (Figure 7). The percentage of surviving cells on the tablets treated with 20 mW and 60 mW parameters varied between 66% and 49% compared to control values. The most significant antimicrobial effect was observed during cultivation with the laser-treated tablets with 100 mW, where the viable cells were 37%. These findings are also confirmed by SEM observations, which revealed significant morphological damage to the bacterial cells, as well as alterations in cell size. The experiments were carried out two times, and the resulting data was used to calculate the average values. Mean values are given with standard deviations of ≤10%. Statistical analysis was performed using OriginPro 6.1.

**Figure 8:**
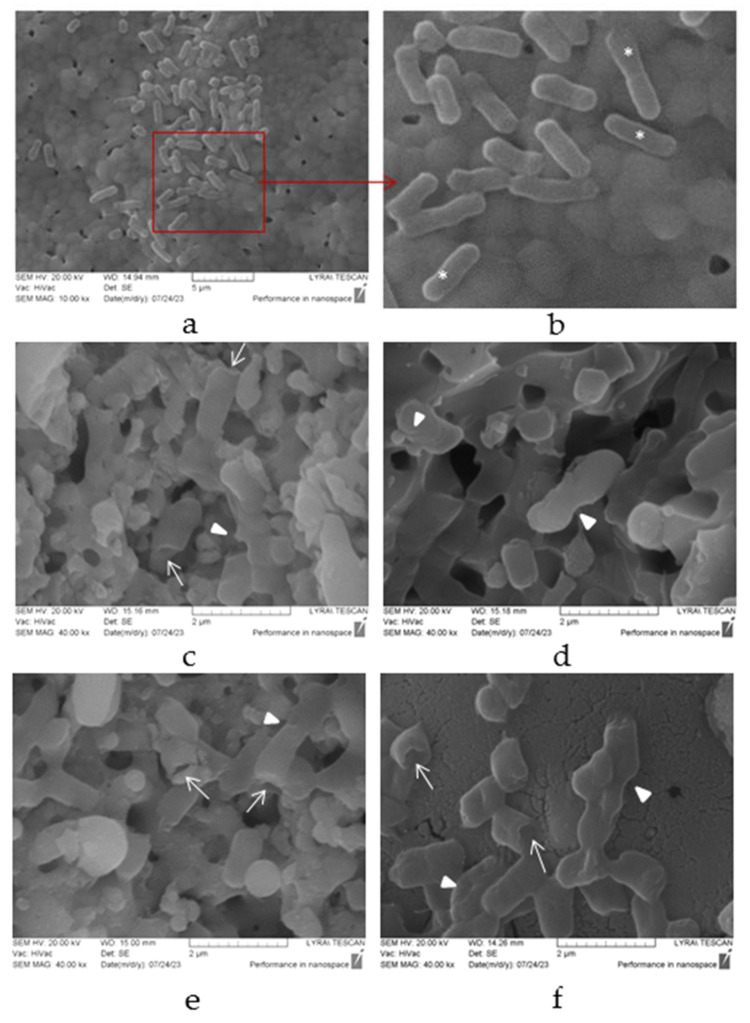
Scanning electron microscopy micrographs of *E. coli* 25922 strain, cultivated on laser-treated ceramic tablets: (**a**,**b**) control group (untreated tablets); (**c**) laser power (P) 20 mW, (**d**) 60 mW and (**e**,**f**) 100 mW and scanning velocity V = 1.5 mm/s. Designations: white stars—normal cells; white arrows—unipolar indentation; white triangles—structural invaginations. Bars = 2–5 µm.

SEM images of the *E. coli* cell morphology (Figure 8) demonstrate the surface topology and morphological changes in bacterial cells resulting from contact with the treated surfaces. We observed significant changes in the morphology of the bacterial cells after 24 h of cultivation in the presence of laser-treated ceramic tablets (Figure 8).

It was shown that in the control probe, where the tablets were not treated with laser, the bacterial cells presented with normal morphology typical of the species. They appeared as rod-shaped cells with rounded ends and intact cell walls. Some cells in the population also showed the formation of bacterial septa, indicating the stage of cell division (Figure 8a,b). However, after the laser treatment, notable morphological changes were observed in the bacteria. The different laser parameters used in the treatment led to the deformation of the cell wall, resulting in the presence of infolds and grooves on the bacterial cell surfaces. These deformations potentially caused a loss of cellular integrity. Additionally, a portion of the bacterial cells became shorter in length, and their surfaces displayed unipolar indentations or the appearance of holes (Figure 8c–f). 

## 4. Discussion

Calcium phosphates that have undergone ion modification are non-stoichiometric compounds in which some of the ions that build up the crystal structure have been replaced by others. The closer the admixture ions are in charge and chemical affinity to the parent ion, the easier the substitution. In this study, Mg/Zn double-doped β-TCP was prepared by a biomimetic approach that includes synthesis of amorphous precursor in a medium of simulated body fluid in the presence of valine as an organic additive. The selected concentrations of Zn (3 mole %) and Mg (7 mole %) are higher than the corresponding concentrations measured for human bones (up to 2.5 mole % Mg and up to 0.16 mole % Zn [36]) in order for the as-prepared material to act as a reservoir for Mg and Zn ions to maintain physiological extracellular concentrations. Our previous studies on the behavior of calcium phosphates substituted with Mg-only or Zn-only showed that they did not change the biocompatibility of the materials in a wide concentration range [37] and in contact with different simulated body fluids, released the ions step by step into the solutions [38,39]. Electrolyte environment of simulated body fluid that is similar to the inorganic part of blood plasma, provides additional inclusion of Cl^−^, Na^+^ and K^+^ ions in the composition analogous to the composition of human bones and teeth [1,2]. Valine was chosen to mimic the organic biomolecules of blood plasma. Included in the composition of the initial modified body fluids (see Section 2.1), the valine forms complexes with Ca^2+^ and Mg^2+^ ions in Solution 2 (Table 1) and with Zn^2+^ ions in Solution 3 (Table 1) reducing the activity of free cations analogous to organic components in the blood plasma [40]. Since the calcium phosphate salts are poorly soluble, HPO_4_^−^ ions from Solution 1 (Table 1) are associated with free metal ions during the synthesis procedure. The precipitation of ion-modified amorphous calcium phosphate leads to stepwise decomposition of the metal-valine complexes in order to obtain new portions of free metal ions and thus to their depletion in the solution. The released valine is washed away with the washing water. 

The precipitation of amorphous calcium phosphates (Figure 1a) is consistent with the notion that kinetic rather than thermodynamic conditions determine the precipitation of amorphous calcium phosphate, as opposed to the thermodynamically stable HA, which we found previously [41] in systems with one doping ion and only simulated body fluid as the medium by precipitation. Both Mg and Zn, together (in this study) or separately [41], increase the stability of amorphous calcium phosphate and stimulate its transformation into Mg/Zn-substituted β-TCP (Figure 1). The contraction of unit cell obtained by Rietveld refinement (Table 2) confirms the substitution of Ca by Mg and Zn in the structure of β-tricalcium phosphate. 

The results of laser-processing (Mg/Zn)-β-TCP showed the ability of fs laser sources to obtain highly accurate laser–matter structuration that can be implemented to functionalize the surface of brittle ceramic scaffolds by precisely adjusting the patterning geometry. The design of surface structures was influenced by the need to create reproducible surface features, by formation of textured surfaces with an impact on the behavior of bacterial cells.

By creating surface textures with increasing laser power and velocity, keeping constant the hatch sizes of 50 µm, formation of deeper patterns with increased surface roughness was observed (Figure 4d). It can be hypothesized that the bacterial cells would not be able to adhere, since the surface could not offer anchoring possibilities due to the laser-created roughness, which varied from 4.9 µm to 9.4 µm (Figure 4a–d). This would prevent the formation of multilayers in the bacterial population and subsequent biofilm formation, because of the additional patterning since the bacterial cells would not be able to overcome the gaps between the laser-carved areas. The textured surfaces consist of peaks and valleys (Figure 3), which can serve as a trap for bacteria. In the experiments, the maximum ablation depth that was achieved was approximately 10 µm regardless of the applied laser power, which could be explained with the development of partial shielding of the incoming laser pulse from the laser-generated plasma. This feature makes patterning conditions reproducible. From the cross-sectional confocal microscopy images, the ablated zones possess a conical form, which enlarges with increasing scanning velocities and applied laser power.

The data obtained from the analysis of an antimicrobial effect reveal that it increases with increasing laser power (P) (Figure 7 and Figure 8). The lowest cell viability accompanied by deformation of the cell wall was obtained at P = 100 mW and scanning velocity V = 1.5 mm/s, which provide optimal surface roughness. Analysis of the *E. coli* cell viability (Figure 7) and morphology (Figure 8) give significant information on existing antimicrobial activity. Such promising results are a reasonable premise for expanding further antimicrobial research in order to improve the properties of ceramic tablets.

## 5. Conclusions

Tablets of Mg (8.44 mol %) and Zn (2.63 mol %) double-doped β-TCP were prepared by biomimetic precipitation of amorphous calcium phosphate in media of simulated body fluid enriched with Mg^2+^ and Zn^2+^ ions and in the presence of valine as an organic additive, followed by step-wise calcination up to 1000 °C. 

Femtosecond laser structuring was successfully applied to the prepared substrates without monitoring changes in the ceramic microstructure. The laser modification induced selective material removal, thus causing a gentle modification of the irradiated zone of the brittle ceramic material. All structures obtained were highly and precisely reproducible and controlled by the precise selection of the applied laser parameters. The complementary results obtained by the SEM and roughness analyses, as well as by the XRD and Raman spectra, clearly demonstrate that the femtosecond laser surface structuring of the (Mg/Zn)-β-TCP pellets is a contactless, finely controlled method that allows precise surface micromorphology design and structuring without significantly affecting the crystallinity and phase of the ceramic material. Moreover, the laser-based patterning method adopted in this study, in combination with additional doping with Mg or Zn, represents a novel approach that was applied directly to enhance the antimicrobial activity of TCP biomaterial. The results demonstrate the potential of femtosecond laser radiation to tune laser-generated structures by texturing, as an alternative therapy for controllin the antibacterial effect of the ceramic surface.

## Figures and Tables

**Table 1:** Composition (mmol·L^−1^) of the conventional and modified simulated body fluids and the buffer solution.

Components	SBFc [32]	Solution 1	Solution 2	Solution 3	Buffer Solution
Na^+^	141.9	141.9	141.9	141.9	100
K^+^	3.0	501	3	3	
Mg^2+^	1.5	0	58.4	1.5	
Ca^2+^	2.5	0	374	2.5	
Zn^2+^	0	0	0	12.2	
Cl^−^	143	143	1008	167	97.5
SO_4_^2−^	0.5	0.5	0.5	0.5	
HCO_3_^−^	4.2	4.2	4.2	4.2	
HPO_4_^2−^	1.0	250	0	0	
Valine		512	512	512	512
pH	7.2–7.4	8.0–8.2	8.0–8.2	6.5	8.0–8.2

**Table 2:** Unit cell parameters for (Mg/Zn)-β-TCP heated at 1000 °C.

Sample	*a* [Å]	*c* [Å]	V, [Å^3^]
β-TCP [35]	10.4352 (2)	37.4029 (5)	3482
This study	10.3238 (1)	37.2624 (6)	3439

## Data Availability

The data presented in this study are available in this article.
